# What is the time cost of exercise? Cost of time spent on exercise in a primary health care intervention to increase physical activity

**DOI:** 10.1186/s12962-020-00209-9

**Published:** 2020-03-18

**Authors:** Lars Hagberg, Stefan Lundqvist, Lars Lindholm

**Affiliations:** 1 Centre for Health Care Science, Faculty of Medicine and Health, Örebro University, P.O.Box 1324, 701 13 Örebro, Sweden; 2 Department of Health and Rehabilitation, Unit of Physiotherapy, Institute of Neuroscience and Physiology, Sahlgrenska Academy, University of Gothenburg, Gothenburg, Sweden; 3 Centrum för Fysisk Aktivitet Göteborg, Svangatan 2B, Region Västra Götaland, 416 68 Göteborg, Sweden; 4 Unit of Epidemiology and Global Health, Umeå University, 901 87 Umeå, Sweden

**Keywords:** Exercise, Time cost, Cost-effectiveness, Voluntary time, Physical activity, Primary health care

## Abstract

**Background:**

In health care interventions aimed at increased physical activity, the individual’s time spent on exercise is a substantial input. Time costs should therefore be considered in cost-effectiveness analyses. The aim of this study was to estimate the cost of time spent on exercise among 333 primary health care patients with metabolic risk factors receiving physical activity on prescription.

**Methods:**

Based on a theoretical framework, a yardstick was constructed with experience of work (representing claim of salary as compensation) as the lower anchor-point, and experience of leisure activity forgone due to extended exercise time (no claim) as the higher anchor-point. Using this yardstick experience of exercise can be valued. Another yardstick was constructed with experience of cleaning at home in combination with willingness to pay for cleaning as the lowest anchor-point.

**Results:**

The estimated costs of exercise time were between 14 and 37% of net wages, with physical activity level being the most important factor in determining the cost. Among sedentary individuals, the time cost was 21–51% of net wages while among individuals performing regular exercise it was 2–10%. When estimating the cost of time spent on exercise in a cost-effectiveness analysis, experience of exercise, work, leisure activity forgone, and cleaning at home (or other household work that may be relevant to purchase) should be measured. The individual’s willingness to pay for cleaning at home and their net salary should also be measured.

**Conclusions:**

When using a single valuation of cost of time spent on exercise in health care interventions, for employed participants 15–30% of net salary should be used. Among unemployed individuals, lower cost estimation should be applied. Better precision in cost estimations can be achieved if participants are stratified by physical activity levels.

*Trial registration* The study was conducted as a survey of existing clinical physical activity on prescription work, and was approved by the Regional Ethical Review Board in Gothenburg, Sweden (ref: 678-14)

## Background

Inadequate physical activity (PA) is associated with increased risk of disease and premature death [1, 2]. Support for increased PA is often used in health care as treatment or prevention, and is generally assumed to be effective and cost-effective [3]. Cost-effectiveness analyses can be performed from different perspectives, but in general a societal perspective is recommended [4, 5]. From this perspective, the individual’s time spent on exercise is a substantial input and should be considered in analyses. In this article, the term “exercise” is used to mean leisure time PA for enjoyment or health, rather than for useful reasons such as transportation or PA during work time.

Even though there is no satisfactory evidence for the time cost of exercise, it is common to simply assume a time cost and include it in the analysis. The use of exercise cost was described in a review of cost-effectiveness of PA interventions [6], but also in later articles, such as cost-effectiveness of physical activity on prescription (PAP) in a Swedish context [7, 8]. Hatziandreu [9] was an early pioneer in this field, suggesting that exercise time should be valued at full wages for those who dislike exercise, at half wages for those who are neutral, and at no cost for those who like exercise. However, it is possible and desirable to make empirical estimates of the time cost of exercise. We have previously suggested a theoretical framework and a measurement method [10]; the framework is summarized below.

### Theoretical framework

Time is usually regarded as an economic resource which everyone holds in the same fixed quantity. Individuals are assumed to allocate their time to different activities in different proportions with the aim of maximizing their utility. Time-dependent utility cannot be stored, and so can only be replaced by utility from activities that can be performed at the same moment.

The value of time is considered to be equal to the utility an individual receives from an activity [4]. Utility of activities can be divided into two parts; utility during the performance of the activity (utility in use) and utility after the activity is performed (utility in anticipation) [11]. This is important when it comes to time spent on exercise, which may be motivated by both enjoyment (utility in use) and better health (utility in anticipation).

In cost-utility analysis, health-related utility in anticipation should be expressed in terms of quality-adjusted life years (QALY) [12]. However, utility in use cannot be measured by QALY, because it is not sensitive to enjoyment, and so the only possible solution is to monetize it [12]. When health gains in anticipation are expressed in QALY, the monetized cost of time is only affected by utility in use.

As a theoretical rule, the utility in use is lower in work than in leisure activities. We accept and spend time in work because we receive salary as compensation, and the level of salary can be assumed to indicate the difference between utility in use of lost leisure activity and utility in use of work. At least in theory, we allocate time between work and leisure in order to maximize our utility, meaning that the utility of the last hour of leisure is equal to the utility of the last hour of work including salary. Based on this observation, a yardstick can be constructed with utility in use of work as the lowest anchor-point and utility in use of leisure activity forgone as the highest anchor-point. The yardstick can be monetized using the amount of salary necessary to make the two points equal with respect to utility level. The cost of the loss of utility in use when spending leisure time on exercising can thus be calculated. Another approach to creating the yardstick is based on purchase of different kind of home services, which means buying time for more leisure activities and thus gaining utility equivalent to the difference between the experience of homework and the experience of leisure activity. This second yardstick can be monetized by the willingness to pay (WTP) for home services.

This measurement method was used as a basis to gather data for estimating the cost of exercise time in an intervention in primary health care in Gothenburg, Sweden. This intervention involved the PAP in primary health care, individualized for patients with metabolic risk factors, with the main aim of the intervention being to increase these patients’ PA level.

## Methods

### Aim

The primary aim of this study was to estimate the cost of time spent on exercise among patients with metabolic risk factors receiving PAP. A secondary aim was to investigate individual factors associated with differences in the cost of time spent on exercise.

### Study population

This study was based on an observational follow-up study of PAP treatment in primary health care, using 6-month follow-up data. The study population consisted of 444 patients from 15 primary health care centers in Gothenburg, Sweden, included during 2010–2014. The study design and medical results are presented in detail elsewhere [13]. The inclusion criteria were: being physically inactive, having at least one component of the metabolic syndrome present, receiving PAP treatment, and understanding the Swedish language.

Of the 444 patients who received the PAP intervention, 368 participated in the 6-month follow-up, and 333 of these filled in the questionnaire on experience of activities. The mean age was 58 years (range 27–84), and 54% were female. The most common reason for the PAP prescription were overweight/obesity (88%) and hypertension (79%) (Table 1).

**Table 1:** Characteristics of participants at the 6-month follow-up (n = 368)

Age in years	58.0 (SD: 10.9)
Sex	
Female	198/368 (54%)
Male	170/368 (46%)
Country of birth	
Sweden	312/363 (86%)
Other	51/363 (14%)
Social situation	
Living alone	135/356 (38%)
Living with partner	205/356 (58%)
Living with other	16/356 (4%)
Employment	
Employed	182/349 (52%)
Retired	123/349 (35%)
Other	44/349 (13%)
Education	
Elementary grade	69/360 (19%)
Upper secondary school	131/360 (36%)
University college	160/360 (45%)
Tobacco	
Smokers	34/359 (9%)
Non-smokers	229/359 (64%)
Ex-smokers	96/359 (27%)
Health status	
Overweight/obesity	311/355 (88%)
Hyperglycemia	127/354 (36%)
Hypertension	283/358 (79%)
Hyperlipidemia	197/356 (55%)
Musculoskeletal disorders	53/353 (15%)
Other	151/353 (41%)

Dropout was related to sex (more female), musculoskeletal disorders, diastolic blood pressure and quality of life. No significant differences between followed up and dropouts were seen for age, socioeconomic factors, tobacco, and all other risk factors/diseases [13].

### Intervention

Personnel at the primary health care centers prescribed an individually tailored PAP to each patient, based on a dialogue with the patient using the principles of motivational interviewing [14–16]. Important factors for the content in the prescription were the patient’s preferences regarding level of PA and different kinds of PA. The patients were offered individual support for 6 months.

### Measurements

**Fig. 1:**
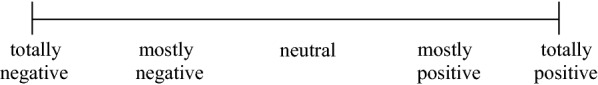
Rating scale for experience of exercise, leisure activity forgone, work, and cleaning

*Cost of time spent on exercise* was measured according to a previously-developed method [10]. This consisted of two main measurements: (1) identification of the leisure activity forgone due to extended exercise time, and (2) rating of the experience of time spent on exercise, work, and the leisure activity forgone on a graphic rating scale (see Fig. 1). It was stressed that only the experience during the activity should be judged. In addition, experience of cleaning at home was measured. The experience of each activity was transformed to a scale running from 0 to 100, assumed to correspond to utility in use.

*Physical activity level* was measured with the Saltin–Grimby Physical Activity Level Scale (SGPALS), which assesses leisure time PA during the past year on four different levels [17].

1. Sedentary—mostly reading, television, computers, cinema, or other sedentary activities.
2. Moderate exercise—walking, cycling, or other activity for at least 4 h a week.
3. Regular exercise and training—running, swimming, tennis, badminton, gymnastics, or other similar exercise for at least 2–3 h a week.
4. Vigorous training and competition—vigorous training and competition in running, cross country skiing, swimming, or football several times a week.

These levels have been validated against measures such as metabolic risk factors [18, 19], and the SGPALS has been published in an updated Swedish form [20].

*Body weight* and *body height* were measured by health care personnel. *Body mass index (BMI)* in kg/m^2^ was calculated from the measured weight and height.

### Estimation of costs of the time spent on exercise

In estimating costs of the time spent on exercise, the following interpretations of measurements were made:

1. When the experience of exercise was graded higher than the leisure activity forgone, the value of experience of exercise was set to the same as for the leisure activity forgone (no time cost).
2. When the experience of exercise was graded lower than both the activity forgone and work, the value of experience of exercise was set to the same as for work (time cost similar to salary).
3. When the experience of exercise was graded in between the experience of work and that of the leisure activity forgone, the value of experience of exercise was estimated by its position on the scale relative to the positions of work and leisure activity (with the time cost on a scale running from 0 to 100% of salary).

As an example, suppose that given a scale of 0–100, an individual valued the experience of work at 20 and that of the leisure activity forgone at 80. If experience of exercise was valued at 20 (the same as work), net salary would be needed as compensation for reaching a utility level equivalent to 80, while a valuation of exercise equal to 80 would mean that no compensation was needed and a valuation of 50 would represent a need of half net salary as compensation.

As will be shown in this article, many individuals rate their experience of work higher than their experience of leisure activity. Experience of work is therefore not always a useful lower anchor-point of the yardstick, and may lead to an overestimation of the cost of time spent on exercise. Hence experience of cleaning was also used as the lower anchor-point of the yardstick. WTP for cleaning was not measured in the actual investigation, but instead it was assumed that the WTP for 1 h of cleaning is between 0.5 and 1.0 h of net salary.

The two methods for estimating the cost of time spent on exercise can be formulated as follows.

When using work as the lower anchor-point,

$$ {\text{C}} = \frac{{{\text{Ul}} - {\text{Ue}}}}{{{\text{Ul}} - {\text{Uw}}}} \times {\text{Hn }} \times {\text{NS}} $$

and when using cleaning at home as the lower anchor-point,

$$ {\text{C}} = \frac{{{\text{Ul}} - {\text{Ue}}}}{{{\text{Ul}} - {\text{Uc}}}} \times {\text{Hn }} \times {\text{WTP}} $$

where *C* denotes cost of time, *U* utility in use, *l* experience of leisure activity forgone, *e* experience of exercise, *w* experience of work, *c* experience of cleaning, *H*_*n*_ number of hours, *NS* net salary, and *WTP* willingness to pay for an hour of cleaning.

### Statistical analysis

Results are presented as mean values and standard deviations. Spearman’s Rank Correlation was used to analyze the impact of personal factors on estimation of cost of time spent on exercise.

## Results

### Experience of activities

Mean experience of exercise (74.3) was somewhat higher than mean experience of leisure activity forgone (70.8). Among employees, work was rated as the activity with highest experience (Table 2). The activities taken into consideration when rating experience of exercise and leisure activity forgone are listed in Table 3.

**Table 2:** Experience of activities

	All (SD)	Employed (SD)
Leisure activity forgone	70.8 (21.5), n = 311	69.2 (19.8), n = 172
Work	74.1 (22.6), n = 223	74.9 (22.7), n = 174
Exercise	74.3 (23.0), n = 332	70.9 (23.4), n = 178
Cleaning	53.8 (25.3), n = 333	50.7 (25.1), n = 179

**Table 3:** Activities

	All (%)	Employed (%)
Exercise		
Walking	48.7	51.8
Biking	6.1	6.1
Gym/indoor workout	23.2	23.8
Gymnastics/aerobics	6.7	5.5
Pool workout	7.0	4.3
Other	8.3	8.5
Leisure activity forgone		
TV	94.9	94.8
Computer	2.0	2.6
Other	3.1	2.6

### Cost of time spent on exercise

When using the yardstick with work as the lower anchor-point, 66% of the participants had higher experience of exercise than of leisure activity forgone, 25% had experience lower than both leisure activity forgone and work, and 9% had experience in between work and leisure activity forgone. The mean time cost was 28.2% (SD: 47.1%) of net salary. When using the yardstick with cleaning as the lower anchor-point, 30% of the ratings were between cleaning and leisure activity foregone, and the estimated cost of time spent on exercise was 13.5–27.1% of net salary (Table 4).

**Table 4:** Cost of time spent on exercise

	Time cost	Experience of exercise		
	Percentage of net salary (SD)	Below yardstick (%)	Above yardstick (%)	Inside yardstick (%)
All, work lower anchor-point of yardstick (n = 268)	28.2% (43.4)	25	66	9
Employed, work lower anchor-point of yardstick (n = 171)	37.1% (46.9)	33	56	10
All, cleaning lower anchor-point of yardstick (n = 308)	13.5–27.1% (40.1)	11	59	30
Employed, cleaning lower anchor-point of yardstick (n = 173)	14.7–29.3% (40.9)	10	55	35

### Factors associated with experience of activities and cost of time spent on exercise

The impact of sex, age, education, BMI, and PA level on cost of exercise time was tested in a bivariate correlation analysis. Sex and BMI had no impact, age and education had some impact, and PA level had a strong impact (Table 5).

**Table 5:** Impact on cost of time spent on exercise

	All, work lower a-p		Employed, work lower a-p		All, cleaning lower a-p		Employed, cleaning lower a-p	
	R	P-value	R	P-value	R	P-value	R	P-value
Sex (M, F)	− 0.04	0.56	− 0.02	0.82	0.01	0.87	− 0.03	0.66
Age	− 0.14	0.02	0.13	0.10	− 0.01	0.81	0.08	0.32
Education	0.15	0.01	0.09	0.25	0.13	0.03	0.09	0.27
BMI	0.11	0.08	0.08	0.29	0.05	0.38	0.08	0.32
PA level	− 0.22	< 0.01	− 0.25	< 0.01	− 0.22	< 0.01	− 0.28	< 0.01

*a-p* anchor-point

When the participants were divided into subgroups by PA level, the costs of time spent on exercise differed considerably between the subgroups (Table 6).

**Table 6:** Cost of time spent on exercise related to PA level

	All, work lower a-p (SD)	Employed, work lower a-p (SD)	All, cleaning lower a-p (SD)	Employed, cleaning lower a-p (SD)
Sedentary	43.6% (49.7)	50.8% (50.2)	21.2–42.4% (46.4)	23.5–47.1% (47.3)
Moderate exercise	29.2% (44.2)	40.1% (47.5)	13.7–27.3% (39.6)	15.0–30.0% (39.9)
Regular exercise and training	7.7% (23.5)	9.6% (26.9)	4.3–8.7% (40.1)	2.4–4.7% (18.9)

*a-p* anchor-point

## Discussion

### Principal findings

We used two measurement methods to estimate the cost of time spent on exercise in a health care intervention to promote PA. The estimated costs were between 14 and 37% of net wages, with a large variation between individuals. PA level was the most important factor determining the value; among sedentary individuals, the time cost was between 21 and 51% of net wages and among individuals performing regular exercise and training it was between 2 and 10%.

### Strengths and weaknesses of the study

The previously-proposed method of estimating cost of the time spent on exercise seems to have shortcomings, due to higher valuation of experience of work than expected. The yardstick based on experience of work will not be adequate for many individuals, as for them, in reality, the estimation of cost of exercise time is based on a higher or lower experience of exercise than experience of leisure activity forgone. For those who rate experience of exercise just below leisure activity forgone, if full net salary is considered as the cost of time spent on exercise, there is likely to be an overestimation of the cost. When instead experience of cleaning at home is used as the lower anchor-point, most values of experience of exercise lower than leisure activity forgone will be inside the yardstick.

This work does have one weakness in that WTP for an hour of cleaning was not measured, and there is room for debate over the assumption of cost corresponding to 0.5–1.0 h of net salary for an hour of paid cleaning. Most people, at least in Sweden, do not pay an hour’s salary for an hour of cleaning. Therefore, using WTP for cleaning corresponding to half net salary seems to be most relevant. To our knowledge, there are no studies on how much people are generally willing to pay for cleaning.

The theoretical construction using experience of work as the basis for valuation does not seem to work well in practice. People’s general experience of work is positive, and experience of leisure time is not always more positive than experience of working hours. In Sweden, working conditions are in general good. How we allocate time between work and leisure time does not seem to match the theory of maximization of utility. In practical use, it seems to be more appropriate to start from the exchange of an hour of housework, such as cleaning, for leisure time. We believe that the maximal WTP to buy an hour of cleaning may be a fairly good measurement of the value of the difference in experience between cleaning and leisure activity. Therefore, in the actual work we prefer the result based on cleaning as the lower anchor-point of the yardstick.

The dropout in the study may have affected the experience of time. Larger drop-out among those with lower quality of life may have resulted in higher experience of time, but it is unclear if it affects the valuation of time spent on exercise.

One might initially think of a method that involves the demand for compensation for exercising instead of the utility in use of leisure activity forgone. However, that valuation is likely to be affected by both utility in use and utility in anticipation, and the demand for compensation will probably be too low. In fact, the participants in the study performed all their exercise without any compensation.

Valuation of time appeared to be different between employed and unemployed individuals. This is in line with an earlier study of the value of travel time, which found that the value was 22.5% lower among the unemployed [21]. The present study, like an earlier study [22], suggests that the opportunity cost (value of leisure activity forgone) may be lower for unemployed individuals. For unemployed individuals in particular, then, it should be more relevant to base the yardstick on WTP for cleaning or similar.

There was a large difference in the costs of time spent on exercise between sedentary individuals and those who regularly performed exercise and training. It is likely that there is a bi-directional causal link, with high time costs leading to less PA. It is also possible to argue that low fitness level gives a less positive experience in the beginning. When starting an exercise regime, the experience seems to be less positive during the first 3 months than after a longer period of exercise [23].

The study was conducted in daily clinical practice among patients in primary care with health problems related to physical inactivity. The cost estimates should therefore be of high relevance for cost-effectiveness analyses of intervention aimed at increased PA in health care. Those who receive PAP are motivated to implement a lifestyle change and may not be representative of the entire patient group with the need for increased PA. In intervention aimed at other sedentary patients, the cost estimation of time spent on exercise in subgroup related to exercise level can help to provide relevant cost of time spent on exercise for those who are least physical active and motivated.

### Strengths and weaknesses in relation to other studies

The most important item from the literature to discuss is the work of Hatziandreu [9] assumed the cost of exercise time to correspond to net salary for those who dislike exercise, half of net salary for those who are neutral, and zero for those who like exercise. The actual study can confirm zero salary (or close to) for those who like exercise, but for those who are neutral or dislike exercise Hatziandreu’s assumption seems much too high. Another common assumption used in cost-effectiveness analyses is that the cost of time spent on exercise is 35% of net salary. This assumption has generally been used in the absence of empirical data, for instance in the analyses of PAP by Feldman [7] and Romé [8]. The assumption may be reasonable for sedentary individuals, but too high for modestly physically active persons. The most important factor making this assumption too high is that the opportunity cost (value of leisure activity forgone) is not as high as net salary.

In comparison with the previous pilot study [10], the present study found that the cost of time spent on exercise was higher despite using the same measurement method; this is probably because the actual study population had poorer health and was less physically active. However, both studies showed a clear difference in the cost of time spent on exercise for sedentary compared to modestly physically active individuals. The development of the measurement method using experience of cleaning instead of work seems to be an advantage.

## Conclusions

When using a single valuation of cost of time spent on exercise in health care interventions, for employed participants 15–30% of net salary should be used. Better precision in cost estimations can be achieved if participants are stratified by PA levels. The results are most relevant to employed individuals. Among unemployed individuals, lower cost estimation should be applied.

For estimation of cost of time spent on exercise in a cost-effectiveness analysis, experience of exercise, work, leisure activity forgone, and cleaning at home (or other household work that may be relevant to purchase) should be measured. In addition, individuals’ WTP for cleaning at home and their net salary should be measured. This will allow estimation of the currently most accurate cost of exercise time.

## Data Availability

The study data are available from the corresponding author on reasonable request.

## Abbreviations

BMI: Body mass index
PA: Physical activity
PAP: Physical activity on prescription
QALY: Quality-adjusted life-years
WTP: Willingness to pay
